# South West Surgical Club

**Published:** 1973-07

**Authors:** 


### South West Surgical Club

The South West Surgical Club held its 1972 Autumn Meeting in Bristol at the Bristol Royal Infirmary and Southmead Hospital. During the meeting some clinical and scientific papers were presented and members toured the Radiotherapy Centre at the Bristol Royal Infirmary. The following papers were presented:—

### PROBLEMS IN THE MANAGEMENT OF PRIMARY PELVIC HYDRONEPHROSIS:

J. B. M. Roberts

This condition has been shown to present at any age after birth and may be discovered at autopsy having been symptomless during life. It may be bilateral in 50% of patients and all the available evidence seems to be that it is a progressive condition. There is a significant risk of infection and stone or tumour formation and treatment should be aimed at avoidance of these complications and prevention of damage to the kidney. A plea was made for relatively early surgery. It was pointed out that relief of symptoms very frequently followed the accepted conservative operations, whilst the kidney could be preserved if not improved. If surgery is not undertaken, then it is importan to carry out a life long follow up on the patient as this condition had proved, in some cases, to be the cause of the patient's death.

### RENAL FUNCTION FOLLOWING URETERIC OBSTRUCTION ASSESSED BY ISOTOPE RENOGRAPHY:

R. H. Jago

Fifty-nine patients with ureteric obstruction were divided into 2 groups, determined by the severity of the insult received by the affected kidney. In each renogram the slope of the second phase of the curve of the affected kidney was expressed as a percentage of that of the normal kidney, and termed the 'function index'. The transit time for the affected kidney was expressed as a multiple of that for the normal kidney and termed the 'transit index'.

Initial renograms taken shortly after admission showed a greater loss of function and prolongation of transit time following major insults than following minor insults, but this distinction disappeared in follow-up renograms. Fifty per cent of the patients whose obstruction had been relieved showed a permanent loss of function in the affected kidney after an average interval of 1 year. The excretory urogram failed to detect loss of function demonstrated by the renogram in 44% of cases.

It was concluded that there is a relatively high and so far largely undetected permanent loss of renal function following temporary ureteric obstruction, and that much of this damage occurs in the initial phases of the obstructive episode, when pelvic pressures are high.

### OBJECTIVE ASSESSMENT OF ELECTRICAL STIMULATION FOR THE TREATMENT OF INCONTINENCE:

C. D. Collins

The urethral response to electrical stimulation from external electrodes has been measured in order to establish the duration of the sphincteric response and secondly to see if any objective change resulted from symptomatically successful electrical treatment. Two methods have been used. The first employs a cantilever force gauge sensitive to the squeeze of the urethral sphincter. The second is a continuous flow pressure profile method which enables measurements of both urethral length and pressure to be taken under a variety of different test conditions (Collins, 1972).

It was found that stimulation from both anal plug electrodes and vaginal electrodes almost always caused a short term response from the urethral sphincter. For this reason an intermittent stimulator system was designed and constructed. The stimulator is used with a vaginal electrode in the treatment of stress incontinence. Each patient is taught to carry out pelvic floor exercise both with and without the concurrent use of the stimulator.

Of 14 patients treated, 5 are now cured and 5 greatly improved 6 months after treatment was discontinued. Pressure profiles taken from this group of 10 patients showed a significant ($p.<.01$) decrease in the shortening of the urethra which occurs on stress.

Collins, C. D. (1972). Observations on the Effect of Electrical Stimulation. Proc. Roy. Soc. Med. **65**, p. 832-833.

### PROGNOSIS IN CARCINOMA OF THE OESOPHAGUS

R. H. R. Belsey

Carcinoma of the oesophagus has acquired the evil reputation of a neoplasm which inevitably leads to the death of the patient in great misery and distress. Palliative surgery has much to offer. Palliation is adequate only when the patient can eat and drink a full normal diet till death occurs from metastases or unrelated causes.

The only worthwhile palliation is a single stage resection and reconstruction of the malignant oesophagus. This procedure was carried out in 322, or 74%, of 434 cases of squamous cell carcinoma. 109 patients were over 70 and 16 over 80 years of age. The overall operative mortality rate was 26%.

The quickest, simplest technique for reconstruction is indicated. In this series a one stage high intrathoracic or cervical oesophago-gastric anastomosis following the oesophagectomy proved to be the method of choice.

12% of the entire series, including the operative deaths, survived 5 years or longer and the average survival period after operation was 26 months. For well differentiated growths without lymph node involvement, the average survival was 65 months. For other form of treatment, such as intubation, the survival time was only 4 months.

Palliative resection and reconstruction can, therefore, offer the patient the opportunity of a further 2 years of normal swallowing. This is acceptable palliation. The basic and essential problem is early diagnosis; the average survival is then extended to more than 5 years. Every patient with any form of dysphagia, irrespective of age, should be oesophagoscoped.

### SERUM GASTRIN IN DUODENAL ULCER AND THE RESPONSE TO SELECTIVE VAGOTOMY:

B. G. Clendinnen and Caroline J. Owens

It has been widely accepted that gastrin levels are low in patients with duodenal ulcers because of antral inhibition by excess acid.

This study of 33 patients with duodenal ulcers and 12 normal controls compared the response of serum gastrin to a protein stimulus, the relationship between intragastric pH and serum gastrin levels and the effects of selective vagotomy on serum gastrin.

Blood was taken for gastrin measurement before and 10, 30, 45 and 60 minutes after an oral Bovril stimulus. The intragastric pH was measured synchronously with a glass pH electrode.

Gastric acid and serum gastrin responses to insulin-induced hypoglycaemia were also measured in ulcer patients. Six months after selective vagotomy the responses to Bovril and insulin stimulation were re-assessed.

There was a direct relation between serum gastrin and intragastric pH in controls ($r=0.786$; $P<0.001$). No such correlation existed in patients. Gastrin levels $>300$ pg/ml occurred at $pH<1.5$ in patients.

Serum gastrin levels rose significantly 30, 60 and 90 minutes after insulin. Selective vagotomy abolished this response but fasting and protein-stimulated gastrin levels were increased.

It is concluded that after oral protein, gastrin levels are greater in duodenal ulcer patients than in controls. High gastrin levels are encountered at low pH in ulcer patients suggesting a degree of autonomy of the gastric antrum. Autonomy is partial as there is a rise in gastrin levels after gastric denervation. Antral denervation abolishes the rise in serum gastrin in response to vagal stimulation.

### SOME ASPECTS OF CROHN'S DISEASE:

L. R. Celestin

Crohn's disease is of unknown aetiology and has no specific therapy. It must be treated in terms of the pathology found and in the light of the disability it causes. At present it is an incurable disorder and at best surgery is palliative. Each patient presents a different problem so that no constant policy can be evolved. This makes any scientific and prospective study very difficult. Diagonosis requires the multi-disciplinary assessment of radiologist, pathologist, physician and surgeon.

An inflammatory exudate characterises the lesion and leads to gross protein loss and causes diarrhoea. This may also be attributable to stasis enteritis, bile-salt irritation, increased peristalsis and intolerance of some food substances.

Thus replacement therapy, supportive treatment and anti-diarrhoeal drugs have priority of place. Steroids are the most useful drugs in stemming the protein loss.

It must be emphasised that the commonest cause of mortality is abdominal sepsis following surgery; that perforation and cancer are rare; that obstructive symptoms respond well to conservative treatment; that life-threatening indications for surgery are few, while surgery should be embarked upon with care and must be highly selective.

A series of cases was presented and examples taken from this to emphasize the aspects mentioned above.

### THE ABDOMINAL REPAIR OF HIATUS HERNIA:

M. G. Wilson, I. S. Bailey and J. B. Penry

The results of surgical treatment of reflux oesophagitis during the period 1958-1970 are reported.

From 1958-1966 a "Simple Repair", i.e. repair of the hiatus and reconstruction of the oesophago-gastric angle was done. From 1966-1970 a Fundoplication was substituted. About half the Simple Repairs also had vagotomy and pyloroplasty, and some had cholecystectomy for associated stones.

Of 97 patients: 70 were assessed at a special follow-up clinic and 11 on their out-patient records, one died postoperatively, 4 died subsequently from unrelated causes and 11 have not been assessed. At the follow-up clinic all patients were categorised 1-4 clinically and 1-4 radiologically: 1 and 2 in each group being good and 3 and 4 poor.

In the entire series 92% had a good clinical result and 78% were good radiologically. Comparing the clinical results of Simple Repair with Fundoplication, 92% in both groups were good, but radiologically 97% of the Fundoplications were good compared with 64% of the Simple Repairs.

Two of 44 Simple Repairs have required further surgery for recurrent oesophagitis. There has been no recurrent oesophagitis in the Fundoplication group, but dysphagia has been a problem requiring re-operation in one and repeated bouginage in 3 others. Post-prandial discomfort due to difficulty in belching also occurs. Vagotomy has not had a beneficial effect, rather, the group without vagotomy were superior both clinically and radiologically.

It is concluded that Simple Repair of hiatus hernia by the abdominal route has given good clinical results in 92% followed up from 6 to 12 years. Neither fundoplication nor vagotomy have improved these results and both can add complications.

### NEONATAL PERFORATIONS:

A. G. McPherson

Twenty four cases encountered at Southmead Hospital in fifteen years are reviewed. Twelve cases were premature or small, ten had respiratory problems at birth.

Thirteen different aetiological factors relating to mother or baby were found. The commonest causes were grangrenous volvulus neonatorum and fibro-cystic disease each of which preceded perforation in 5 cases. Maternal hydramnios was present in four, Rhesus haemolytic disease in five, but in three only was exchange transfusion implicated. Fifteen cases had meconium peritonitis from perforation occurring antepartum or shortly after birth; only five of these had fibro-cystic disease.

The usual presentation was with bilious vomiting distension and failure to pass normal meconium. Four cases had large abdomens noted at birth. Plain abdominal X-rays were of great value in diagnosis. Pneumo-peritoneum was diagnostic but gaseous bowel distension was absent from early films.

Seventeen cases were treated by surgery, seven were considered unfit for operation because they were too small or too ill from serious associated conditions. Six cases have survived since 1967 while two only survived in the earlier years.

### INSULIN RESISTANCE IN BURNS:

R. W. Pigott

Under stress the anabolic hormone insulin is suppressed, allowing glucose to be mobilised and converted into energy. In some circumstances such as major burns the adrenal hormones which suppress insulin release continue to be secreted for days or weeks after injury. During this time the phenomenon of insulin resistance develops when very high levels of circulating immuno-reactive insulin are found in response to 25 g. glucose infusions. However, this insulin does not appear to function as normal insulin and prolonged catabolism results from the ineffective response to it. A gross negative nitrogen balance exists despite a basic calorie intake of 5,000 to 6,000 calories per day.

This catabolic state is exacerbated by inadequate peripheral perfusion following an insidious fall in blood volume and infection at the blood/burn interface which produces capillary thrombosis and epithelial loss. Further complications are septicaemia and a "sick cell syndrome" when potassium is lost into the urine and extracellular sodium enters the cells. Although serum and urinary sodium concentrations fall, total body sodium is hardly reduced at all. This hyponatraemia is saline resistant.

The catabolic state and hyponatraemia may be corrected by restoring the peripheral circulation with blood and supplementing the calorie intake with 1,500 ml of 50% glucose given with massive quantities of insulin regulated by hourly urine testing and with potassium supplements in the region of 100 to 120 mEq/L. A massive sodium diuresis then occurs in association with a rising serum sodium. Ill-advised saline infusion given to "treat" the low serum sodium may result in a gross excess total body sodium.

The weak, listless, anorexic, somewhat oedematous, peripherally cyanosed patients with reduced bowel sounds typical of this state may be dramatically improved by one or two days treatment on this regime.

### SOME OBSERVATIONS ON THE TREATMENT OF GANGRENE BY SAPHENOFEMORAL BYPASS GRAFT:

R. E. Horton

### THE PLACE OF A RADIOTHERAPY DEPARTMENT IN AN ONCOLOGY UNIT OR SERVICE

R. C. Tudway

The present trends towards the establishment of oncological centres in this country and the contribution of radiotherapy centres to the treatment of malignant disease were reviewed.

It was pointed out that the field covered at present by the radiotherapy centres (i.e. not only the treatment of malignant disease by ionising radiations, but the combination of this treatment with chemotherapy and the collaboration between radiotherapist and surgeon in the combined use of excision and radiation) made the radiotherapy department in a main regional centre a natural focus for an oncological service.

A discussion then followed concerning the proper function and scope of an oncological centre. There was some division of opinion but the general consensus appeared to be that the work of an oncological centre would not include the general acceptance of patients from wide areas of the region, or for that matter, from nearby hospitals. The function of a centre would rather be that of a forum for discussion of problems which would be open to the consultants concerned. It would also be a clearing house for information, and inevitably would be involved in teaching and research, the latter elements being essential stimulants to useful work in the centre.

### THE PLACE OF STAGING LAPAROTOMY AND SPLENECTOMY IN THE MANAGEMENT OF HODGKIN'S DISEASE:

J. Bullimore

The better prognosis in Hodgkin's disease over the past decade has been brought about by improvement in the acuracy of determining the extent of the disease before treatment is commenced; by the use of higher dosage and wider fields with megavoltage radiation in the radical treatment of early disease and the development of more effective chemotherapy for advanced disease.

The Ann Arbor classification is a detailed staging system for Hodgkin's disease, which is widely used. In most centres the treatment of Stages I, II and IIIA is by radical radiotherapy, and Stages IIIB and IV by chemotherapy.

The history, clinical examination, blood picture, liver function tests, liver and spleen scans and lymphogram of the abdominal and pelvic nodes fail to exclude the possible presence of occult disease in many patients. The logical next step is to perform a staging laparotomy and splenectomy. At this operation, ideally, nodes are taken from both common iliac regions, the para-aortic group, splenic pedicle, porta hepatis and from any group of nodes appearing abnormal on the lymphogram. The spleen is removed and a wedge biopsy and three deep needle biopsies of the liver and an iliac crest bone biopsy are taken. Clips are placed at biopsy sites to help in subsequent planning.

In leading centres in the U.S.A. and Europe approximately one third of clinical Stage II and a half of Stage IIIA patients are found to have occult disease. Nearly 100% of Stage IIIB patients have splenic and/or liver involvement. The frequency of occult disease is related to the histological type. Occult disease is more commonly found in lymphocyte depleted and mixed cellular types and is less common in lymphocyte predominant and nodular sclerosis types.

The purpose of staging laparotomy and splenectomy is often an accurate as possible assessment of the stage of the disease and so enables the optimum treatment to be given to the individual patient. It seems that it is justified in all cases except females with Stage 1A disease. These are the only group of patients in which the chance of occult disease being present is so rare as to make the operation unjustifiable.

### TUMOUR GROWTH RATES:

M. Westwood

Exponential models have frequently been used for tumour growth analysis, both in the extrapolation of clinically observed growth and in the comparison of growth rates under experimental conditions.

An assumption of cell division with constant doubling time implies exponential growth, which is therefore the *a priori* model for tumour growth. In many cases observed growth patterns have followed this closely, at least over limited periods often until environmental or nutritional factors limited growth. However, in some cases this model has been found to be less satisfactory and so more complicated ones such as the Gompertz have been used.

Examination of the total history of a tumour from a single cell through 40 doublings to about 1,000 grams shows how the observed period of growth is always a small part of the total. Extrapolations outside the observed growth period therefore involves massive assumptions and so can only be carried out in broad terms. Further, there is no evidence that a more sophisticated model such as Gompertz produces any significantly better results.

Comparison of experimentally observed growth rates is often over a limited period during which an exponential model is acceptable. In work on mammary tumours in mice this assumption allowed a powerful statistical method of analytical comparisons to be used which would not have been possible with a more complex growth model.

Until observations can be made over large periods in the total life of a tumour, the basic exponential model is adequate both for any tentative broad extrapolations and for growth rate comparisons.

